# Extending the Eclipse^TM^ AcurosXB output factor table for small field radiosurgery

**DOI:** 10.1002/acm2.13877

**Published:** 2022-12-31

**Authors:** Ning Genevieve Wu

**Affiliations:** ^1^ Department of Radiation Medicine Oregon Health and Science University Portland Oregon USA

**Keywords:** Eclipse small field dosimetry, Small Field Output Factor Measurement

## Abstract

**Purpose:**

To investigate the necessity of extending the output factor table (OF Table) of the Varian Eclipse^TM^ Treatment Planning System for small field stereotactic radiosurgery (SRS) and stereotactic body radiosurgery (SBRT) treatments.

**Methods:**

A new AcurosXB 15.6 beam model was created in the Eclipse Beam Configuration, which is identical to the one that has been used in the clinic with a default 3 × 3 cm to 40 × 40 cm OF Table, except the OF Table in the new model was extended to cover the range from 1 × 1 cm to 40 × 40 cm. 80 small square and rectangular output factors were measured on a Varian TrueBeam utilizing a Standard Imaging Exradin W2‐1×1 scintillator detector, inside a PTW BeamScan water tank with 95 cm SSD at 5 cm depth. Cerenkov contamination was corrected using a rectangular field method (2 cm × 15 cm field). Nine Radiosurgery plans with primary jaw setting ranging from 0.7 cm to 2.0 cm were evaluated by both beam models. The monitor unit (MU) differences between the two beam models were calculated for identical 3‐dimensional (3D) absolute dose distributions. Output factors, measured versus Eclipse calculated, were compared down to 0.5 × 0.5 cm primary jaw setting.

**Results:**

For the 6FFF beam, the difference between the two beam models was ∼ 6% for 1 × 1 cm jaw settings and 4% at 1.5 × 1.5 cm, with the extended OF Table requiring higher MUs for the same dose prescription and same 3‐dimensional isodose distribution. For the 6MV beam, the corresponding difference is ∼7.5% for 1 × 1 cm, 5% for 1.5 × 1.5 cm, and 3% for 2 × 2 cm jaw settings, with the extended OF Table requiring higher MUs. For jaw settings smaller than 1 × 1 cm, measured dose can be considerably smaller than Eclipse predicted dose, even with the OF Table extension. This is reflected by the fact that the output factor for 0.5 × 0.5 cm, calculated via Eclipse external beam, was more than 30% greater than that measured for both 6FFF and 6MV beams.

**Conclusions:**

Eclipse does a satisfactory job for primary jaw sizes down to 2 cm. For jaw settings smaller than 1.5 cm, the OF Table in Eclipse should be extended to improve the dose calculation accuracy.

## 1 INTRODUCTION

Radiosurgery is a highly effective treatment modality in the management of brain lesions and compliments the traditional craniotomy.^1^ It is a low risk outpatient procedure with fast recovery; good for small lesions near critical anatomical structures, and for older patients.^1^ The advent of radiosurgery has heralded a revolution in the management of brain metastasis, and it has also provided an effective alternative to the management of benign tumours or neurological conditions, such as acoustic neuroma, meningioma, trigeminal neuralgia, arteriovenous malformation, pituitary adenomas, and it is the preferred treatment option for certain conditions.^1^

Linac‐based radiosurgery has considerably broadened the application of radiosurgery and has made radiosurgery an available choice for patients at a majority of radiation oncology centres in the United States. Varian Linear Accelerators, including Truebeam, Edge, and Trilogy are the most widely used platforms in Linac‐based radiosurgery, while Eclipse^TM^ is one of the most widely used treating planning systems for Varian Linac‐based radiosurgery treatment planning and treatment delivery.^2^

**FIGURE 1:**
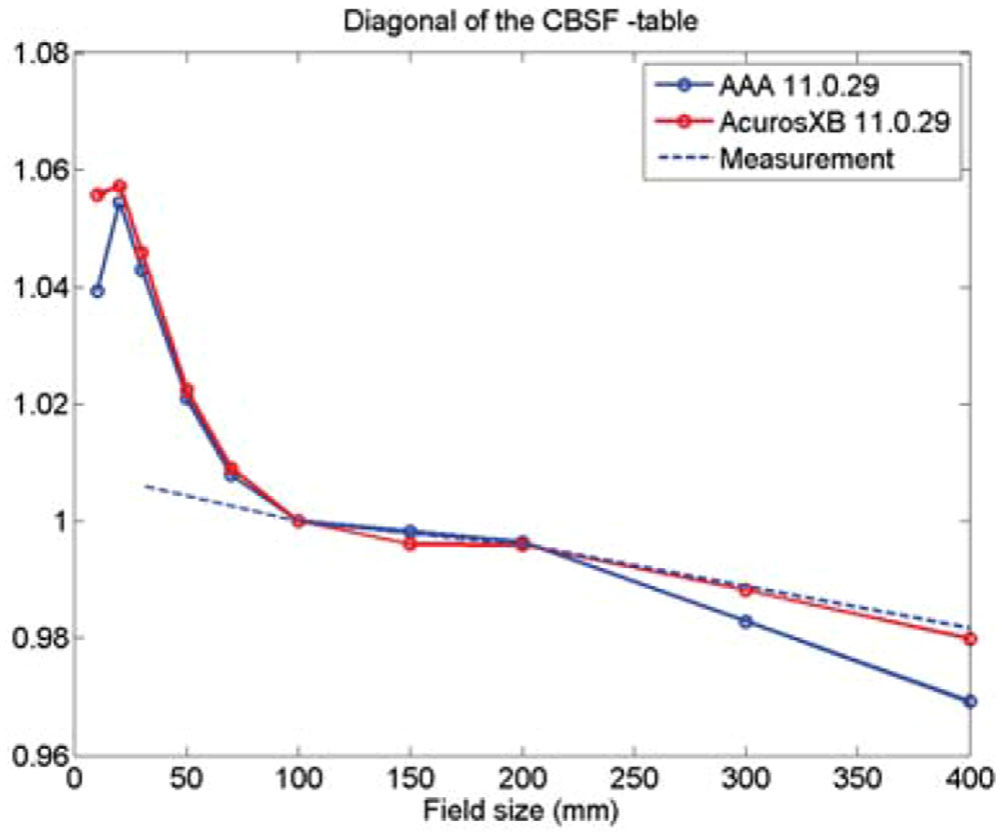
Collimator backscatter factors for symmetric and square jaw‐limited fields for a Varian C‐series machine (quoted from Ref. 3 with permission).

The Eclipse^TM^ treatment planning system was originally designed for planning treatments with conventional, broad photon fields.^3^ Enhancements required for small fields treatment planning have been added incrementally in the newer Eclipse versions.^3^ Even today, the data collection (percentage depth doses, profiles, output factors) for Eclipse commissioning does not include field sizes smaller than 3 × 3 cm.^4^ Prior to 2013, as shown in Figure 1, the Eclipse calculated collimator backscatter factors (CBSF) started to deviate from measurements at a field size of 10 × 10 cm; at 1 × 1 cm, the calculated CBSF markedly differed from that of measured data.^3^

CBSF is related to the Output Factors through Equation (1)^4^

(1) $$CBSF\;\left(X,Y\right)=\;\;\frac{OFref}{OF\left(X,Y\right)}*\frac{D\left(X,Y\right)}{Dref}$$

where *OF_ref_* is the output factor at a reference field size; *OF (X,Y)* is the output factor for field size X, Y; *D(X,Y)* is dose calculated by Eclipse at a reference point for field size X, Y, ignoring CBSF; and *D_ref_* is the dose calculated by Eclipse for reference condition, ignoring CBSF.^4^

In 2013, Varian made a major improvement in Eclipse small field calculation accuracy through the following approaches: (1) set the primary jaws at 3×3cm or larger, and utilizing MultiLeaf Collimators (MLC) to define the field apertures; and (2) In Eclipse Beam Configuration, manually adjusting the effective target spot sizes in both X and Y directions, so that the calculated dose would match the measured absolute dose levels for small MLC‐delimited apertures, as well as matching the measured penumbra width. With such an approach, Varian states that Eclipse can accurately handle MLC delimited small fields down to 5 × 5 mm.^3^

If the primary jaw smaller than 3 × 3 cm are used clinically, it would be important to include accurate output factor measurements for the minimum field size used when configuring the model.^3^ Eclipse does not require depth dose or profiles to be smaller than 3 × 3 cm for jaw settings less than 3 × 3 cm, only the output factors; in fact, depth dose or profiles smaller than 2 × 2 cm are not used by model configuration algorithm even if they are included in the input data.^4^

The accuracy of small field dosimetry has been a challenge, due to the loss of lateral charged particle equilibrium on the beam axis; partial occlusion of the primary photon source by the collimating devices on the beam axis; detector volume averaging; the detector perturbation of the particle fluence in the medium, etc.^5^ Amid these challenges, the accurate determination of the small field output factors (OF) has been one of the most important components in small field dosimetry.

In 2017, the International Atomic Energy Agency (IAEA) and American Association of Physicists in Medicine (AAPM) published an International Code of Practice for Dosimetry of small static fields used in external beam radiotherapy,^5^ which introduced the concept of output correction factor, and provided concrete and clear guideline for clinical physicists on small fields dosimetry. Mamesa et al.^6^ demonstrated that the application of IAEA/AAPM TRS 483 reduces the discrepancy of calculated MU among three beam commissioning datasets using three different detectors, for 6MV photon beams and Acuros XB algorithm in Eclipse Treatment Planning Systems (TPS). Lechner et al.^2^ did a multinational investigation to compare output factors calculated by TPS versus output factors obtained through measurement, and found modern TPS beam models generally overestimate the OFs for small fields.^2^ The deviations increase with decreasing field sizes, particularly so for Eclipse.^2^ Sendani et al.^7^ investigated the effect of small field OFs on the TPS calculation accuracy for three different TPS algorithms (AAA, Acuros XB, CCC) and three different field settings (jaw, MLC, and jaw/MLC). They concluded that when jaw or jaw/MLC defined fields are smaller than 2 × 2 cm, using the corrected output factors improves the accuracy of dose calculation in all three algorithms.^7^ When field sizes are smaller than 1 × 1 cm,^2^ the robustness of the dose calculation algorithm becomes important.^7^ Larraga‐Gutierrez et al.^8^ measured MLC defined field output factors in a TrueBeam STx following TRS‐483^5^ recommendations, using four radiation detectors, to evaluate the AcurosXB algorithm in Eclipse TPS. The backup jaws were set at 0.5 cm larger than the MLC shaped fields. They concluded that by optimizing the focal spot size to minimize the difference between the calculated and measured dose profiles, the calculation accuracy of AcurosXB can be improved to an acceptable range. Behinaein et al.^9^ compared Eclipse calculated PDDs, profiles, and output factors for both AAA and AcurosXB algorithms with Monte Carlo simulations as well as Exradin W1 scintillator measurements. A Varian TrueBeam was used for deliveries. They found W1 scintillator measurements agree well with Monte Carlo (MC) simulations, and that AcurosXB had better agreement with MC and measurements than AAA did.

The measurement results of small field output factors are detector dependent.^5^ Many examples have been published showing large discrepancies between the ratio of readings measured with different types of detectors for a particular beam compared with the actual ratio of absorbed dose to water values.^5^ Akino et al.^10^ collected 6 and 10 MV beam data of Varian TrueBeams from multiple institutions, measured with multiple different small field detectors, and found the inter‐institutional variability was large ‐ especially at the field size of 5 × 5 mm. After applying the output correction factors per IAEA/AAPM TRS‐483, the variation reduced from more than 15% to within ±5% from the median field output factors.^10^

It is possible that even after applying the output correction factors provided by IAEA/AAPM TRS‐483, the inherent inaccuracy of each detector still may not have been fully corrected. This has been shown in Akino's paper (±5% after the application of the output correction factors),^10^ and is also the author's personal experience. In light of this, with the recent availability of the plastic scintillator detectors developed by Standard Imaging Inc, particularly its newest product W2‐1 × 1 with a smaller dimension of 1 × 1 mm, compared with the previous W1's 1 × 3 mm, W2‐1 × 1 has become the best choice to minimize the inherit detector errors without the need to apply output correction factors.

In this work the author presents: (1) the results of primary jaw defined small field output factors, measured on a Varian TrueBeam, with a Standard Imaging Exradin W2‐1×1 Scintillator detector in a PTW water tank with the application of auto beam centring; (2) the difference in the dose calculation results for small jaw‐delimited radiosurgery treatments using an OF Table extension compared with no OF Table extension in Eclipse AcurosXB beam configuration; (3) the comparison between W2‐1×1 measured OF and Eclipse AcurosXB calculated OF; and (4) under what circumstance the Eclipse AcurosXB output factor table needs to be extended.

## 2 BACKGROUND

A Varian TrueBeam 2.7 was commissioned for Radiosurgery and SBRT treatments, paired with the Eclipse^TM^ treatment planning system, Acuros algorithm, and Mobius3D as the second check. The output factor tables in Eclipse^TM^ Beam Configuration were initially the default 3 × 3 cm to 40 × 40 cm.

Target sizes as small as 5 mm have been treated with the radiosurgery protocol using this TrueBeam/Eclipse combination. Many such small lesion radiosurgery treatment plans were handled with dynamic conformal arcs (DCA), with primary jaw settings less than, equal to, or larger than 1cm. Small jaw settings were used in order to generate a tight margin around the small targets, and to minimize dose to the surrounding normal tissue. In the process of doing so, discrepancies of 5–10% for jaw sizes 1 cm or smaller were noted between measured point doses during patient specific QA and planned doses, with planned dose consistently being higher than measured dose. Discrepancies on the order of those seen by others^11^ were also noted between the treatment plan and the secondary calculation software, Mobius 3D (Varian, Palo Alto, CA, USA). The discrepancies of the former prompted the author to investigate the efficacy of extending the Eclipse output factor table.

## 3 MATERIALS AND METHODS

### 3.1 Design comparison method in Eclipse

A new beam model was created in Eclipse Beam Configuration using Acuros 15.6. The new model was identical to the one used clinically for patient treatment calculations, except the new model's output factor table was extended to cover the range from 1 × 1 cm to 40 × 40 cm; compared with the original default 3 × 3 cm to 40 × 40 cm.

Nine Radiosurgery plans from four previously treated patients, 35 dynamic conformal arcs in total, with primary jaw settings ranging from 0.7 cm to 1.5 cm, were evaluated by both beam models; that is, the model with the output factor table extension and the model without output factor table extension.

**FIGURE 2:**
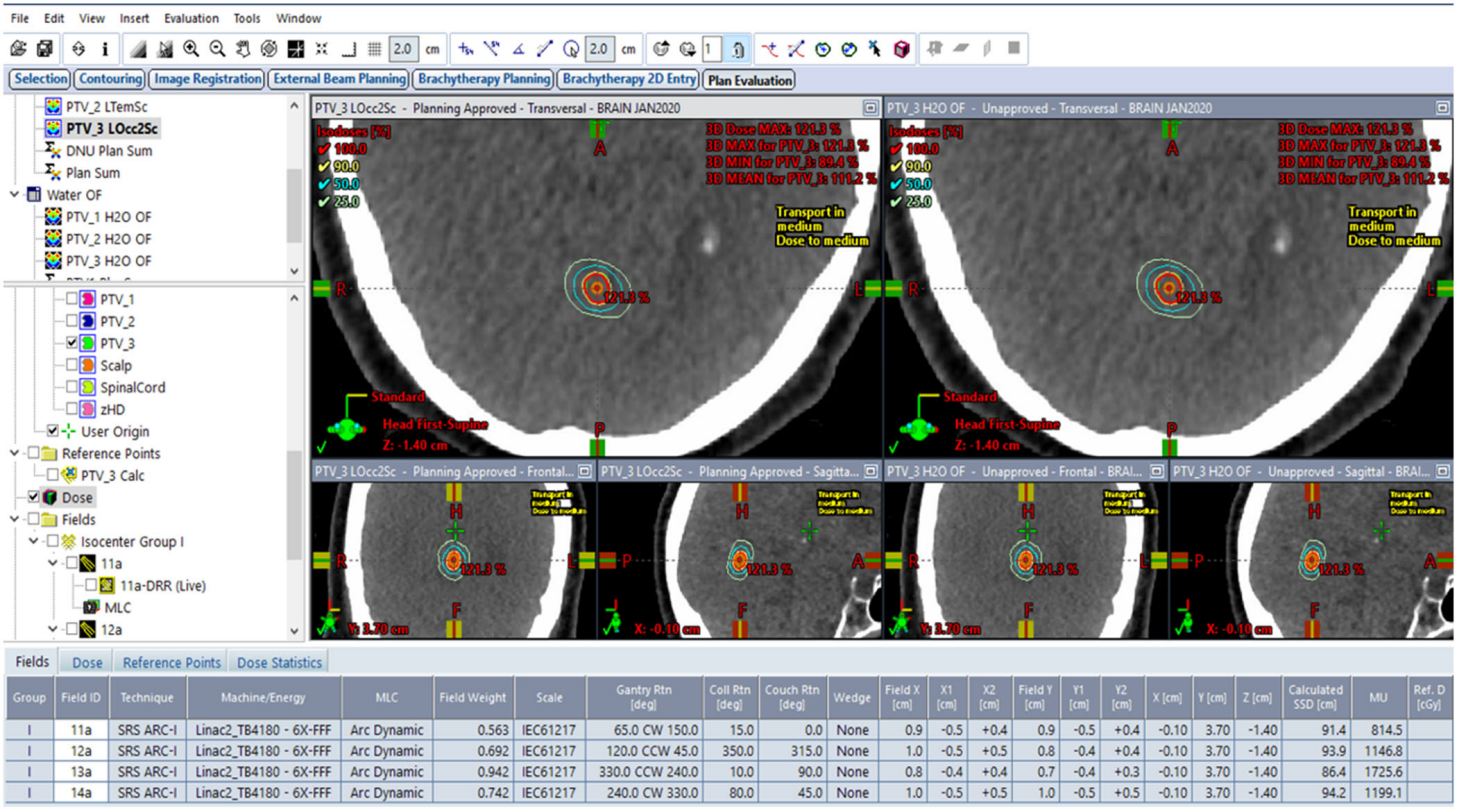
3D absolute isodose distributions were kept the same between the two beam models. The one on the left was original treatment plan without OF table extension; the one on the right is the plan re‐calculated with OF table extension.

**FIGURE 3:**
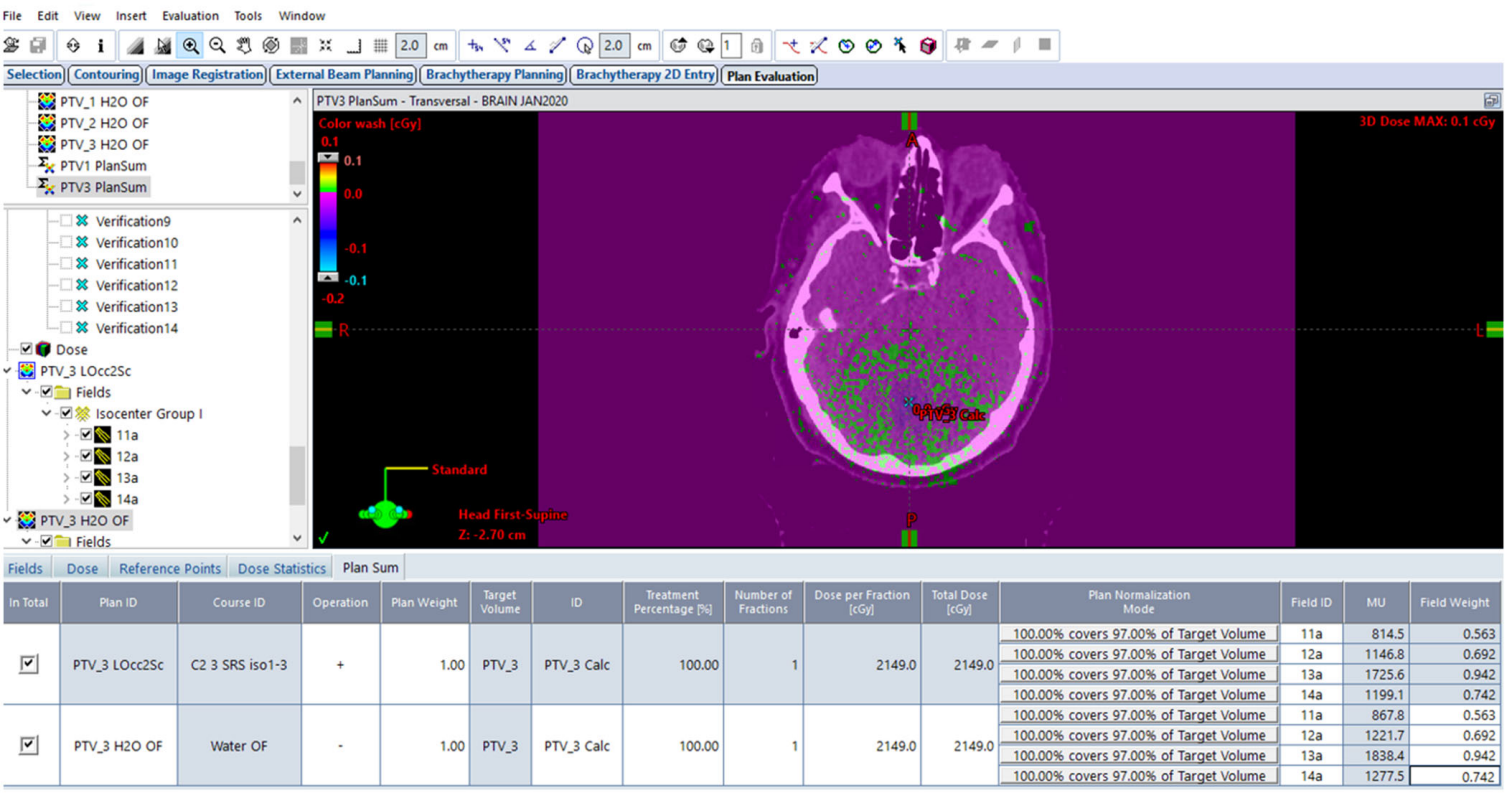
Screenshot of the 3D dose calculated with output factor table extension was subtracted from the 3D dose calculated without output factor table extension. Purple region showed zero remaining dose, while green dots are locations with <0.1 cGy remaining dose. The MU differences between the two identical plans are showing in the column second to the last.

The prescriptions and the absolute three‐dimensional (3D) isodose distributions were kept constant between the two beam models (Figure 2), and the difference between the required monitor units for the same 3D absolute isodose distribution were calculated (Figure 3).

Figure 3 presents the dose difference between the two plans in Figure 2. The purple region shows zero dose, while the green dots are locations with less than 0.1 cGy remaining dose. However, the MUs required to generate the identical absolute isodose distribution were much higher when the output factor table was extended, as shown in Figure 3 (PTV_3 LOcc2Sc is the plan without OF table extension, while PTV_3 H_2_O OF is the same plan with OF table extension).

### 3.2 Patient specific plan point dose measurements

Patient specific plan point doses were measured for each individual field of each patient plan, using Sun Nuclear Corporation's StereoPhan holding an SRS MapCheck (with SunPoint2 diode detectors) insert, Sun Nuclear StereoPhan holding a PTW PinPoint 3D Chamber insert, or Sun Nuclear ArcCheck with a PTW MicroDiamond detector insert.

### 3.3 The small fields output factor measurement

The 80 additional small field square and rectangular output factors, 1 × 1 cm up to 1 × 40 cm as well as 2 × 1 cm up to 2 × 40 cm and the inverse, as shown as the top two rows and left two columns in Tables 1 and 2, for 6MV and 6FFF were measured with Standard Imaging Exradin W2‐1 × 1 scintillator detector.^12^ The geometrical field size defined by primary jaws was chosen, based on how Eclipse uses its output factor table for dose calculations.^4^

**TABLE 1:** The extended Eclipse output factor table for 6FFF beam

Field size FX [mm]											
Field size FY [mm]		10.0	20.0	30.0	50.0	70.0	100.0	150.0	200.0	300.0	400.0
	10.0	0.739	0.782	0.796	0.802	0.804	0.807	0.808	0.809	0.809	0.810
	20.0	0.794	0.853	0.866	0.879	0.884	0.888	0.890	0.892	0.893	0.893
	30.0	0.804	0.867	0.896	0.914	0.922	0.928	0.932	0.934	0.937	0.937
	50.0	0.810	0.881	0.914	0.940	0.952	0.962	0.969	0.972	0.975	0.976
	70.0	0.814	0.888	0.925	0.955	0.970	0.982	0.991	0.996	1.000	1.001
	100.0	0.816	0.892	0.933	0.967	0.985	1.000	1.012	1.018	1.024	1.025
	150.0	0.818	0.896	0.938	0.975	0.996	1.013	1.029	1.038	1.046	1.048
	200.0	0.819	0.897	0.941	0.979	1.001	1.020	1.038	1.049	1.058	1.060
	300.0	0.821	0.900	0.944	0.983	1.006	1.028	1.048	1.061	1.072	1.075
	400.0	0.822	0.900	0.945	0.985	1.009	1.031	1.052	1.065	1.078	1.080

**TABLE 2:** The extended Eclipse output factor table for 6 MV beam

Field size FX [mm]												
Field size FY [mm]		10.0	20.0	30.0	40.0	50.0	70.0	100.0	150.0	200.0	300.0	400.0
	10.0	0.708	0.757	0.769	0.773	0.776	0.778	0.781	0.783	0.784	0.785	0.786
	20.0	0.768	0.832	0.848	0.854	0.861	0.866	0.870	0.874	0.877	0.878	0.880
	30.0	0.777	0.849	0.880	0.891	0.899	0.908	0.915	0.920	0.923	0.926	0.926
	40.0	0.781	0.856	0.892	0.906	0.915	0.927	0.936	0.943	0.946	0.950	0.951
	50.0	0.784	0.861	0.901	0.917	0.928	0.942	0.952	0.961	0.966	0.970	0.971
	70.0	0.788	0.870	0.913	0.932	0.946	0.963	0.977	0.988	0.995	1.001	1.003
	100.0	0.792	0.876	0.924	0.946	0.962	0.983	1.000	1.015	1.024	1.032	1.035
	150.0	0.794	0.881	0.932	0.956	0.974	0.999	1.020	1.039	1.052	1.063	1.067
	200.0	0.796	0.885	0.936	0.961	0.980	1.007	1.031	1.053	1.067	1.082	1.086
	300.0	0.799	0.887	0.941	0.967	0.987	1.016	1.043	1.069	1.086	1.104	1.110
	400.0	0.800	0.890	0.943	0.970	0.990	1.020	1.048	1.076	1.095	1.113	1.119

For this investigation, the detector was placed inside a PTW BeamScan water tank with a 95 cm source to surface distance at 5 cm depth, with the optical fibre oriented perpendicular to the axis of the radiation beam. BeamScan's auto‐centring technique was used to ensure that the W2‐1×1 was placed at the true radiation isocentre. With scintillator measurements, Cerenkov emissions are created when the optical fibre connecting the scintillating material to the photodiode is irradiated. The amount of Cerenkov light produced is proportional to the irradiated length of the fibre. To correct for the Cerenkov component of the signal, a two‐channel method is used.^13^ This method requires two measurements in which the dose to the scintillator remains constant, but the length of the optical fibre inside the irradiated field changes. The difference in the signal between these two configurations is used to correct the Cerenkov emissions.^13^ For this investigation, the Cerenkov correction was performed using the rectangular field rotation method described in the MAX SD user manual.^12^

Measurements of at least three stable readings were collected for both maximum and minimum fibre configurations. A field size of 2 × 15 cm was chosen for the rectangular field rotation method. For the output factor measurements, three readings were taken for the 10 × 10 cm field size, and two readings were taken for all other small field sizes since the readings agreed with each other to the 4^th^ decimal point.

The W2‐1 × 1 measured small fields output factor results were compared with those measured by a PTW Semiflex 3D ion chamber at the fields sizes of 4 × 4 cm and 5 × 5 cm, under the same setup conditions (95 cm SSD and 5 cm depth of measurement in the same water tank). For 6FFF, the difference in OF between the two detectors was smaller than 0.6%; for 6MV, the corresponding difference was 1.13% at 4 × 4 cm, and 0.88% at 5 × 5 cm. For both energies, output factors measured with the Semiflex 3D were slightly larger.

## 4 RESULTS

### 4.1 Output factor measurement

Table 1 shows the extended output factor table for the 6FFF beam. The top two rows and the left two columns in pink background are the newly added data measured with W2 1×1. The data with light blue background are the original data from the commissioning. Table 2 shows the corresponding output factors for 6MV beam. The output factors in the same column or row, when plotted against the jaw size, should form a smooth curve.

### 4.2 Percentage difference in Acuros calculation: Of table extension versus no extension

Table 3 shows the percentage difference in calculated MUs for both 6FFF and 6MV beams, with and without output factor table extension. The 6FFF data includes nine dynamic conformal arc plans and 35 total arcs, while the 6MV data includes seven plans and 29 total arcs. The plans calculated with the extended OF table require much higher MUs for the same plan, same prescription, and the same 3D absolute isodose distribution as shown in Figure 3.

**TABLE 3:** Percentage difference in calculated MUs, with and without OF table extension. Patient specific point dose measured data are included for the 6FFF fields

Energy	Jaw size (cm)	# of arcs	Average % difference in MU	Max % difference in MU	Average % difference, measured versus calculated (no OFT extension)	Max % difference, measured versus calculated (no OFT extension)
6 FFF	0.7–0.9	9	6.13% ± 0.01%	6.14%	8.47% ± 1.7%	10.68%
	1.0	5	6.13% ± 0.01%	6.14%	5.24% ± 1.24%	6.84%
	1.0–1.1	7	5.86% ± 0.15%	5.99%	4.97% ± 1.56%	6.53%
	1.1–1.2	5	5.41% ± 0.10%	5.83%	4.32% ± 1.47%	6.69%
	1.2–1.4	4	4.92% ± 0.22%	5.26%	3.46% ± 0.9%	3.85%
	1.4–1.6	4	4.12% ± 0.26%	4.50%	2.94% ± 0.51%	3.24%
6 MV	0.7–1.0	11	7.55% ± 0.06%	7.57%		
	1.1–1.2	2	6.97% ± 0.32%	7.19%		
	1.2–1.4	3	5.83% ± 0.17%	5.96%		
	1.4–1.7	3	5.07% ± 0.13%	5.17%		
	1.7–1.8	2	4.53% ± 0.01%	4.53%		
	1.9–2.2	8	2.98% ± 0.12%	3.03%		

Rx and 3D isodose distribution were kept identical between plans calculated with and without OF Table extension.

From the data in Table 3, there appears to be a direct correlation between jaw size (2^nd^ column) and the percentage MU difference (4^th^ column). For 6FFF, the difference between the two beam models is around 6% for 1 × 1 cm jaw setting, about 4% at 1.5 × 1.5 cm jaw setting, with the extended output factor table requiring higher monitor units for the same radiation dose prescription, and the same 3‐dimensional absolute isodose distribution. For 6 MV beam, the difference between the two beam models is around 7.5% for 1 × 1 cm, 5% for 1.5 × 1.5 cm, and 3% for 2 × 2 cm jaw settings, respectively, with the extended output factor table requiring higher monitor units for the same radiation dose prescription, and the same 3D absolute isodose distribution.

### 4.3 Acuros calculation versus patient specific point dose measurement

In order to examine the agreement between dose calculated by Acuros versus point dose measured for individual patients, the nine 6FFF patient treatment plans were re‐calculated using the new model with the extended output factor table, but keeping the MUs the same as the original plans calculated without OF Table extension. Table 4 provides these results for field sizes equal to or less than 1 × 1 cm, which show that the extended OF Table substantially reduced the difference between measurements and the Eclipse calculations. The patient specific point dose data were only available for 6FFF beams since measurements had been made only for SRS patients, and all SRS patients were treated with 6FFF beams.

**TABLE 4:** Comparison between Eclipse calculated dose and measured point dose for fields at or below ∼1cm

Plans	Beam	Appro. jaw setting	measured results cGy	Acuros calculated (no OFT ext)	% diff mea versus Acuros (no OFT ext)	Acuros Calculated (w OFT extension, same MU)	% diff mea versus Acuros (w OFT ext)
Measurement: StereoPhan holding SRS Mapcheck, couch zero							
1	03a	0.8 × 0.8	428.41	467.83	−8.43%	433.40	−1.15%
	04a	0.8 × 1	580.60	625.78	−7.22%	579.10	0.26%
	05a	0.8 × 0.9	786.93	878.28	−10.40%	813.00	−3.21%
	06a	0.8 × 0.8	604.92	668.77	−9.55%	620.10	−2.45%
	composite		2400.86	2640.66	−***9.08%***	2445.60	−***1.83%***
2	07a	0.9 × 1	449.46	482.20	−6.79%	432.30	3.97%
	08a	1 × 1	621.90	661.00	−5.92%	593.90	4.71%
	09a	0.8 × 1.1	869.44	959.10	−9.35%	863.10	0.73%
	10a	1.1 × 1.2	736.86	769.90	−4.29%	698.20	5.54%
	composite		2677.66	2872.20	−***6.77%***	2587.50	***3.48%***
3	11a	0.9 × 0.9	449.61	476.80	−5.70%	428.40	4.95%
	12a	0.8 × 1	603.66	662.10	−8.83%	594.60	1.52%
	13a	0.7 × 0.8	839.22	944.90	−11.18%	840.30	−0.13%
	14a	1 × 1	680.69	719.10	−5.34%	647.80	5.08%
	composite		2573.18	2802.90	−***8.20%***	2511.10	***2.47%***
Measurement: StereoPhan with PinPoint chamber 31016 3D, SuperMax, no couch kick							
9	9	1 × 1	306.10	320.30	−4.43%	290.80	5.26%
	10	1 × 1	387.15	419.70	−7.76%	387.00	0.04%
	11	0.9 × 0.9	709.70	760.00	−6.62%	697.90	1.69%
	12	1 × 1.1	725.40	756.90	−4.16%	699.30	3.73%
	composite		2128.35	2256.90	−***5.70%***	2075.00	***2.57%***

Calculations were made with and without OF table extension, keeping the MU constant between the two models.

The extended OF Table model has also shown improved agreement with point dose measurements for larger fields, that is, between 1.1 and 1.5 cm, but the data in this range are less conclusive, partly due to the large uncertainty in the point dose measurements. Guidance is also unclear whether an additional output correction factor should be applied to the SRS MapCheck results for these small fields, similar to TRS‐483 corrections for these diodes.

### 4.4 W2 measurements *versus* eclipse calculation for small field output factors

To take advantage of the W2‐1 × 1 scintillator detector capabilities, and to further the study, output factors smaller than 1 × 1 cm were also measured; these results are compared with those calculated via Eclipse External Beam in Table 5. The measurement was performed at 95cm SSD, 5cm depth in a water tank, with radiation beam auto‐centring applied. The calculation was performed with Acuros 15.6 using the extended output factor table, 1 mm calculation resolution, 95 cm SSD and 5 cm depth.

**TABLE 5:** Output factor comparisons: W2 measurements versus Eclipse^TM^ Acuros external beam calculations with the extended output factor table

	**6FFF Output Factors**			**6 MV Output Factors**		
**Field size (CM)**	Acuros calculated	**W2‐1** × **1 measured**	**Percentage difference**	**Acuros calculated**	**W2‐1** × **1 measured**	**Percentage difference**
10 × 10	1.000	1.000	0.00%	1.000	1.000	0.00%
0.5 × 0.5	0.614	0.456	34.65%	0.567	0.427	32.79%
0.5 × 1	0.666	0.607	9.72%	0.626	0.567	10.41%
0.5 × 2	0.705	0.639	10.33%	0.668	0.598	11.71%
1 × 0.5	0.666	0.579	15.03%	0.657	0.541	21.44%
2 × 0.5	0.694	0.601	15.47%	0.684	0.565	21.06%
1 × 1	0.742	0.739	0.41%	0.712	0.708	0.56%
2 × 2	0.856	0.853	0.35%	0.834	0.832	0.24%
2 × 1	0.784	0.782	0.26%	0.760	0.757	0.40%
3 × 1	0.798	0.796	0.25%	0.773	0.769	0.52%
4 × 1	0.803	0.800	0.38%	0.776	0.773	0.39%
5 × 1	0.805	0.802	0.37%	0.779	0.776	0.39%
1 × 2	0.796	0.794	0.25%	0.772	0.768	0.52%
1 × 3	0.807	0.804	0.37%	0.780	0.777	0.39%
1 × 4	0.810	0.808	0.25%	0.784	0.781	0.38%
1 × 5	0.813	0.810	0.37%	0.787	0.784	0.38%

The 1st number in Field Size represents the X‐jaw, and the 2^nd^ number represents the Y‐jaw.

## 5 DISCUSSIONS

### 5.1 The Exradin W2 ‐1 × 1

The Exradin W2‐1 × 1 scintillation detector has revolutionized small field measurements. Its water equivalence, energy independence, dose rate independence, ability to be used for beam scanning, and its 1 × 1 mm dimension, has made it the best choice for small field measurements in high energy photon and electron beams.^5^, ^12^ Galavis et al. had extensive experimental investigation of W2's parameters such as dose and dose rate linearity, profiles, percentage depth dose, output factors, and temperature response.^14^ Still, care needs to be taken to achieve accurate measurement results. Deriving a correct Cerenkov Light Ratio (CLR) value is crucial; therefore, the method of removing the Cerenkov contamination can make a difference in the accuracy of the measurement results. A good CLR needs to have a large enough difference between the maximum and minimum fibre lengths to reduce the noise; and the maximum fibre configuration should include more fibre than the fibre exposed by the maximum field at any single point. Also, the W2 detector needs to be oriented horizontally in the water phantom, perpendicular to the direction of the radiation beam. Currently no water tank manufactures provide holders specific for W2; but Standard Imaging Inc. provides stem adaptors that match with standard chamber holders, accommodating either a 7 mm or 8 mm diameter chamber stem, so that W2 can be used in a broad variety of water tanks. Early investigators had also used 3D printing technique to create a holder for W2‐1 × 1 in parallel geometry.^14^ In terms of phantoms, a water tank is the best choice for accurately centring the probe in the radiation field. For fields sizes <=1 cm, the accuracy of the detector position becomes crucially important.

### 5.2 The limitation of Eclipse^TM^ for jaw setting smaller than 1 cm

In Table 3, the maximum MU difference is around 6.13% for all jaw settings equal or smaller than 1 × 1 cm; in Table 4, the maximum MU difference is around 7.5% for all jaw sizes of 1 cm or smaller.

Such phenomena are most likely resulted from Eclipse's 1 × 1 cm minimum allowable OF. For jaw settings smaller than 1 × 1 cm AcurosXB relies only on the reduction of phantom scatter but still utilizes the 1 × 1 cm collimator backscatter factor,^4^ and therefore it is not surprising that it fails to calculate doses accurately; this limitation applies to the AAA algorithm as well. Such a fact is also reflected on the last column in Table 3, where the point dose measurement results showed that for jaw settings smaller than 1 × 1 cm the measured dose to the target could be as much as 10% lower than the Eclipse calculation. Note that the maximum calculated difference is 6.14% between calculations using the standard versus the extended OF table.

This fact is further illustrated in Table 5, where, for jaw sizes 1 × 1 cm and above, there is negligible difference between the measured and Acuros calculated output factors. However, if one of the two jaws is at 0.5 cm, there is considerable difference between the two. At 0.5 × 0.5 cm, this difference is greater than 30%, with Eclipse giving much larger output factor values. This means that for jaw settings smaller than 1 × 1 cm, the actual dose delivered for patient treatments can be much lower than what Eclipse calculates.

### 5.3 Future direction

In this work, the fields sizes for the output factor measurements were defined only by the primary jaws, per Eclipse Output Factor Table requirement. To be more closely representing the clinical situations, small field output factors should also be measured for the same aperture sizes including the multileaf collimator (MLC) in‐line with the primary jaws. If significant differences result when the MLC is also included it may become necessary to select an OF Table appropriate to the actual collimation configuration that will be utilized.

## 6 CONCLUSIONS

The Eclipse Acuros algorithm provides a satisfactory dose calculation for multi‐leaf delimited small field sizes down to 0.5 × 0.5 cm, so long as the primary jaw setting remains at 3 × 3 cm or larger. It also does an acceptable job for primary jaw settings down to 2 × 2 cm (within ∼2% for 6FFF beam, and ∼3% for 6 MV beam). Therefore, using the MLC to define the field size while maintain a fixed jaws of 2 × 2 cm for SRS/SBRT treatment planning in Eclipse may be a reasonable approach with acceptable uncertainties.

However, for jaw settings equal to or smaller than 1.5 × 1.5 cm, the output factor table in Eclipse should be extended to improve the dose calculation accuracy.

For jaw settings smaller than 1cm, Eclipse may not calculate dose accurately; therefore, jaw settings smaller than 1cm should be avoided in clinical situations.

## CONFLICT OF INTEREST

No conflicts of interest.
